# Detecting *Alu* Element Insertion Variant in *RP1* Gene Using Whole Genome Sequencing in Patients with Retinitis Pigmentosa

**DOI:** 10.3390/genes15101290

**Published:** 2024-09-30

**Authors:** Hye-Ji Kwon, Beom-Hee Lee, Joo-Yong Lee

**Affiliations:** 1 Department of Ophthalmology, Uijeongbu St. Mary’s Hospital, College of Medicine, The Catholic University of Korea, Uijeongbu 11765, Republic of Korea; 2 Department of Ophthalmology, Asan Medical Center, University of Ulsan College of Medicine, Seoul 05505, Republic of Korea; 3 Medical Genetics Center, Asan Medical Center, University of Ulsan College of Medicine, Seoul 05505, Republic of Korea

**Keywords:** retinitis pigmentosa, RP1, *Alu* element, whole genome sequencing

## Abstract

**Background/Objectives:** *Alu* element insertion in the exon 4 of the *RP1* gene was newly identified through whole genome sequencing (WGS). This was not detected in previous next-generation sequencing (NGS) analysis. We report three cases of Korean retinitis pigmentosa (RP) patients with compound heterozygous variants including *Alu* element insertion in the *RP1* gene, indicating that *Alu* element insertion could be a cause of RP; **Methods:** Among patients diagnosed with RP having variants in the *RP1* gene in the Asan Medical Center, WGS was additionally performed for genetically unsolved cases in previous NGS analysis to detect any presence of *Alu* element insertion. For cases detected to have *Alu* element insertion in the exon 4 of the *RP1* gene, genetic and clinical characteristics were analyzed; **Results**: Among 16 patients with RP, 3 patients were detected to have *Alu* element insertion in the *RP1* gene. *Alu* element insertion in the *RP1* gene was also detected using WGS. It was revealed to be a pathogenic variant. Therefore, *RP1* gene mutation was the confirmed genetic cause of RP for these three cases and genetic counseling was enabled for them; **Conclusions**: *Alu* element insertion in the *RP1* gene could be a genetic cause of autosomal recessive RP patients with compound heterozygous variants. Through WGS, the identification of this pathogenic variant was possible. Confirmation is needed to check the presence of *Alu* element insertion in patients with compound heterozygous variants in the *RP1* gene.

## 1. Introduction

The *RP1* gene is known as a causative gene for retinitis pigmentosa (RP). It is inherited in an autosomal dominant or recessive manner [1,2,3]. Mutations in the *RP1* gene account for 5~6% of genetically identified Korean RP patients [4,5]. Of the identified mutations in *RP1*, most are clustered along exon 4, which is the largest exon^3^. In autosomal dominant RP or autosomal recessive RP, different mutations in exon 4 have been reported, confusing the interpretation of heterozygous mutations [6,7]. Recently, through whole genome sequencing (WGS), an *Alu* element insertion mutation in the exon 4 of the *RP1* gene that was not found in previous next-generation sequencing (NGS) analysis was newly identified [8]. The insertion of a mobile short interspersed element *Alu* in exon 4 of *RP1* resulted in 328 additional nucleotides and a premature termination codon in the *RP1* coding sequence (c.4052_4053ins328, p.(Tyr1352Alafs); *Alu* element insertion), which was reported by recent studies in Japan [7,8,9]. *Alu* element insertion has also been found to be the most frequent variant in Japanese patients with *RP1*-associated retinal dystrophies [10]. However, to the best of our knowledge, studies evaluating clinical and genetic features associated with *Alu* element insertion in Korean RP patients have not been reported yet.

In this study, we report clinical and genetic features associated with *Alu* element insertion in the *RP1* gene of Korean patients with RP. Three patients with autosomal recessive RP and compound heterozygous variants in the *RP1* gene are described, showing that *Alu* element insertion could be a pathogenic variant of RP.

## 2. Materials and Methods

### 2.1. Patients

This study was approved by the Institutional Review Board of Asan Medical Center (IRB No. 2023-0402). Informed consent was obtained from all patients. Among patients clinically diagnosed with RP who underwent preceding molecular diagnosis through targeted NGS in the Asan Medical Center from November 2018 to October 2022, patients with detected variants in the *RP1* gene were identified. Among a total of 25 patients with genetic variation in the *RP1* gene, 9 patients were confirmed to have a pathogenic mutation in the *RP1* gene. All of the remaining 16 patients with autosomal recessive RP had one variant in the *RP1* gene. These 16 patients remained genetically unsolved without a confirmative genetic cause. The presence of *Alu* element insertion in *RP1* was screened with WGS for these 16 RP patients. Familial segregation analysis was carried out for cases with detected *Alu* element insertion.

### 2.2. Analysis of Genetic Variants

Genomic DNA was isolated from peripheral blood samples collected from probands, with or without their patients. We performed targeted NGS using the Ion Torrent S5XL platform (Thermo Fisher Scientific Inc., Waltham, MA, USA) using a panel of 88 genes associated with RP including RP1 [11]. The mean depth of coverage was approximately 500-fold, with 99.2% of coverage being higher than 20-fold. The verification of identified variants for Torrent S5XL sequencing data was waived when the read depth was over 100 reads and the allele frequency was 40 to 60% [12]. The entire process of genome sequencing, analysis, and interpretation was carried out using the RareVision™ platform (Inocras, San Diego, CA, USA). DNA extraction from blood samples was conducted using Allprep DNA/RNA kits (Qiagen, Venlo, The Netherlands). DNA libraries were prepared with TruSeq DNA PCR-Free Library Prep Kits (Illumina, San Diego, CA, USA), and sequencing was performed on the Illumina NovaSeq6000 platform (Illumina, San Diego, CA, USA), achieving an average coverage depth of 30×. The resulting genome sequences were aligned to the human reference genome (GRCh38) using the BWA-MEM algorithm [13]. PCR duplicates were removed with SAMBLASTER [14]. Base substitutions and short indels were called using HaplotypeCaller [15] and Strelka2 [16], respectively. Structural variants (SVs) and transposable elements (TEs) were identified using Delly [17] and MELT [18], respectively. Detected variants were filtered, and their inheritance patterns were evaluated based on Mendelian genetics. De novo mutations were identified, and their effects were predicted. Pathogenicity predictions were further refined using an in-house software that integrates the latest databases. Medical geneticists made the final determination of variant pathogenicity, taking into account the patient’s clinical phenotype and family history.

Confirmation of the *Alu* insertion identified through WGS was achieved using PCR and gel electrophoresis. An optimized PCR-based analysis was conducted to validate the founder *Alu* element insertion in *RP1* as follows. A DNA sample was acquired from the peripheral blood of each subject. Genomic DNA was extracted with a QIAmp DNA blood kit (Qiagen, Hilden, Germany) according to the manufacturer’s instructions. DNA was obtained from 0.2 mL of whole blood, which was treated with SD-containing lysis buffer and protease after purification by spin columns. PCR was performed using a primer set designed to include the site of *Alu* element insertion (forward: 5′-CATGTAACCCCAGTGACACTTT-3′ (chr8:55540260_5554082) and reverse: 5′-GCATTTTCATCCTGAAACTTCCT-3′ (chr8:555402717_55540740)), the extracted genomic DNA, and Taq polymerase (Promega, Madison, WI, USA). PCR involved denaturation at 95 °C for 30 s, annealing at 58 °C for 30 s, and extension at 72 °C for 45 s for 30 cycles. PCR amplicons were subjected to a 2.0% agarose gel electrophoresis with appropriate markers and controls to detect *Alu* element insertion. If *Alu* element insertion was present, the size of the amplicon was 809 base pairs, whereas the size of the amplicon without *Alu* element insertion was 481 base pairs.

### 2.3. Clinical Diagnosis of Retinitis Pigmentosa

All patients underwent ophthalmic examinations including measurements of best-corrected visual acuity (BCVA), slit-lamp examination, and dilated-pupil fundus examination. Multimodal imaging was also conducted, including wide-field fundus photography, optical coherence tomography (OCT), Goldmann visual field test, and full-field electroretinography (ERG) for the clinical diagnosis of RP. Data of a comprehensive clinical history including onset of ocular symptoms, presence of associated systemic symptoms, and family history of diseases were also collected.

## 3. Results

Among the unsolved 16 RP patients with one previously identified variant in the *RP1* gene through targeted NGS, 3 patients were detected with an additional variation, namely *Alu* element insertion in *RP1*. After the novel detection of *Alu* element insertion in exon 4 of the *RP1* gene via re-analysis using WGS, the genetic cause and an autosomal recessive mode of inheritance were confirmed. The genetic basis of RP was undetermined for three patients before resequencing. Therefore, *RP1* gene mutation was concluded as the cause, and genetic counseling was enabled for these three patients.

No patients had a history of other ocular or systemic diseases. All patients were males. The age of onset raged from 10s to 20s and age at clinical diagnosis varied from 16 to 37. Clinical manifestation varied. BCVA at the time of genetic examination in three cases ranged from the best 20/32 to the worst 20/200 (Table 1). The one variant in the *RP1* gene previously identified in different cases included c.4196delG, c.6181del, and c.6181del (Table 1). Genetic information and clinical findings of the three cases are described below in detail.

### 3.1. Case 1

**Figure 1:**
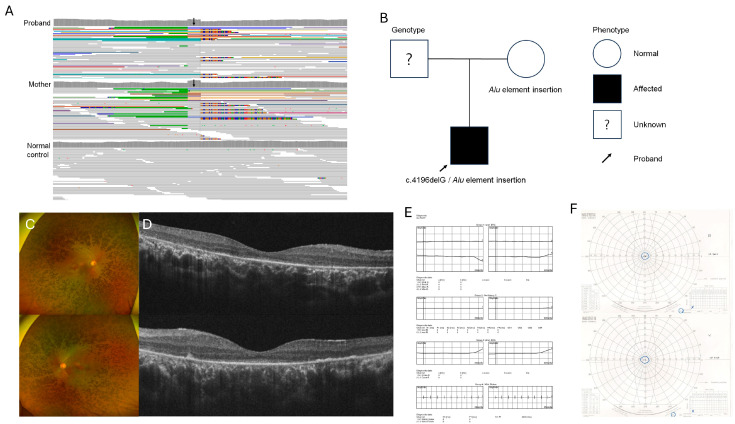
Visualization of *Alu* element insertion by whole genome sequencing (WGS) analysis (**A**). The *Alu* element insertion in the exon 4 was detected in the patient and his asymptomatic mother’s *RP1* gene (black arrow). The pedigree of a 26-year-old male RP patient and his parents (**B**). Clinical findings of wide fundus photograph showed diffuse retinal pigmentation (**C**). Optical coherence tomography revealed disruption of outer segment (**D**). No responses were detected in full-field electroretinography (**E**). Visual field loss preserving central 10° was shown in Goldmann visual field test (**F**).

A 26-year-old male who was clinically diagnosed with RP when he was 16 years old underwent a genetic analysis. One pathogenic variant [c.4196delG (p.C1399 LeufsTer5)] in the *RP1* gene was detected through targeted NGS. Since his parents were both asymptomatic, re-analysis with WGS was planned. The patient was genetically confirmed to have the disease caused by a compound heterozygous variant of c.4196delG (p.C1399 LeufsTer5) and *Alu* element insertion mutation in the *RP1* gene through WGS of the mother (Figure 1A,B). An *Alu* element insertion in exon 4 was inherited from the asymptomatic mother. Since there was no available genetic information from the asymptomatic father, it is unknown where the other variant [c.4196delG (p.C1399 LeufsTer5)] originated from. The patient might have developed an autosomal recessive RP due to a transposable element mutation inherited from the asymptomatic mother. The visual symptom of night blindness started when he was a teenager. The BCVA was 20/32 in both of his eyes. Bony spicules and diffuse retinal degeneration with pigmentation were detected in the peripheral retina, relatively sparing the major vascular arcade (Figure 1C). In the OCT finding, the ellipsoid zone (EZ) was blurred with the disruption of the outer retinal layers, relatively sparing the fovea (Figure 1D). The ERG performed at the time of diagnosis when he was 16 showed flat waves with no detectable responses (Figure 1E). The Goldmann visual field test showed visual filed constriction sparing only the central area within 10° in both eyes (Figure 1F).

### 3.2. Case 2

**Figure 2:**
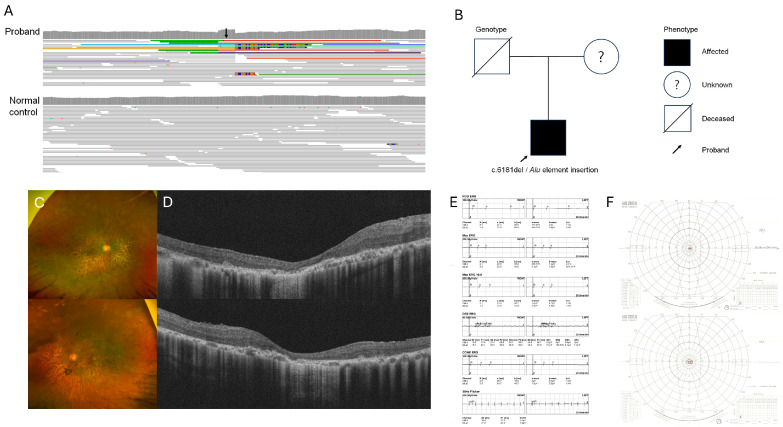
Visualization of *Alu* element insertion by WGS analysis (**A**). The *Alu* element insertion in the exon 4 was detected in the patient’s *RP1* gene (black arrow). The pedigree of a 40-year-old male RP patient and his parents (**B**). His familial genetic information was not available. Diffuse retinal degeneration including macula is shown in wide fundus photograph (**C**). Optical coherence tomography shows disruption of all outer layers (**D**). No responses were detected in full-field electroretinography (**E**). Goldmann visual field test reveals advanced visual field loss sparing only central 5° (**F**).

A 40-year-old male clinically diagnosed with RP when he was 37 had undergone a genetic study. One pathogenic variant [c.6181del (p.Ile2061fs12)] of the *RP1* gene was detected by targeted NGS. The patient’s parents had no history of ocular disease. His familial genetic information was not available. Through additional genetic analysis by WGS, an *Alu* element insertion in exon 4 of the *RP1* gene was detected. Finally, the patient was genetically confirmed to have RP caused by compound heterozygous variants of c.6181del (p.Ile2061fs12) and *Alu* element insertion mutation in the *RP1* gene (Figure 2A,B). The initial onset of decreased visual acuity started in his mid-20s. The BCVA was 20/200 in his right eye and 20/63 in his left eye. Wide-field fundus photography revealed diffuse retinal degeneration with pigmentation involving both peripheral retina and macula (Figure 2C). OCT revealed the disruption of the EZ and the thinning of all the outer retinal layers involving the macula (Figure 2D). The ERG test performed at the age of 37 showed no responses in cone or rod (Figure 2E). The Goldmann visual field test showed visual field loss only sparing the central 5° in both eyes (Figure 2F).

### 3.3. Case 3

**Figure 3:**
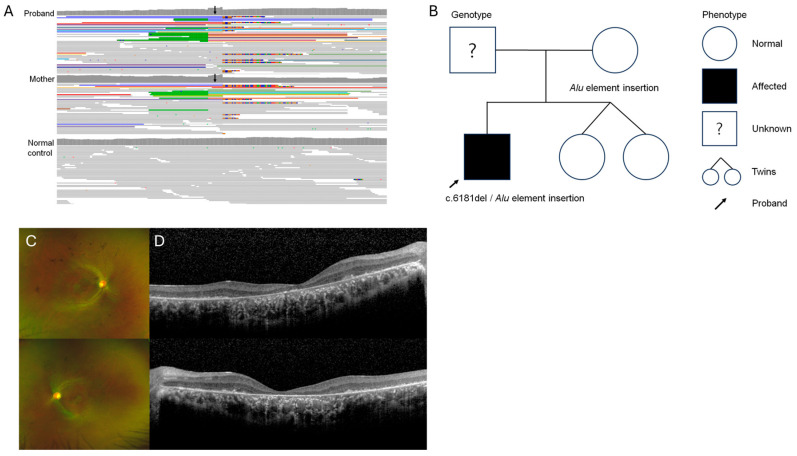
Visualization of *Alu* element insertion by WGS analysis (**A**). The *Alu* element insertion in the exon 4 was detected in the patient and his asymptomatic mother’s *RP1* gene (black arrow). The pedigree of a 27-year-old male RP patient and his parents (**B**). Wide fundus photograph reveals retinal degeneration with bony spicules without involving arcade vessels (**C**). Optical coherence tomography shows foveal atrophy and disruption of all outer layers (**D**).

A 27-year-old male clinically diagnosed with RP underwent targeted NGS at his initial visit to Asan Medical Center. Since one pathogenic variant [c.6181del (p.Ile2061fs12)] of the *RP1* gene was detected with both his parents being asymptomatic, an additional study was performed. Genetic information of his mother was available. The *Alu* element insertion in exon 4 of the *RP1* gene was detected in both the patient and his mother through WGS (Figure 3A,B). The patient was genetically confirmed to have compound heterozygous variants of the *Alu* element insertion inherited from his mother and c.6181del (p.Ile2061fs12) in the *RP1* gene. The age of his symptom onset was in his early 10s. The BCVA was 20/50 in his right eye and 20/200 in his left eye. Retinal degeneration with bony spicules without involving arcade vessels was present in wide-field fundus photography (Figure 3C). OCT revealed the loss of the EZ and the disruption of all the outer retinal layers including foveal atrophy (Figure 3D). Since the initial clinical diagnosis of RP was carried out at another ophthalmology clinic, ERG test and visual field test were not available for this patient.

## 4. Discussion

This study presents three RP patients with a novel pathogenic variant, an insertion of the *Alu* element in the *RP1* gene, that was present in 3 of 16 RP patients with inconclusive results in previous genetic analysis. By detecting the *Alu* element insertion in exon 4 of the *RP1* gene as an autosomal recessive mode of allele through re-analysis using WGS, the genetic cause was clarified for these three cases. Therefore, genetic counseling for patients and their family was enabled. Whole exome sequencing (WES) has been widely adopted in clinical genetics, but it has limitations that WGS can overcome, particularly for rare genetic disorders. WES is limited by the incomplete coverage of coding regions [19] and struggles to detect certain structural variations and variants in non-coding regions [19,20]. In contrast, WGS offers more uniform and comprehensive coverage of the genome, allowing for the detection of a broader range of genetic variations, including complex structural rearrangements and mobile element insertions such as *Alu* elements, which are often missed by WES. *Alu* elements, comprising over 10% of the human genome, can significantly impact gene function and are increasingly recognized as important in rare genetic disorders [21]. While WGS presents challenges in data analysis and interpretation, its comprehensive nature and potential to end diagnostic odysseys for patients with rare genetic disorders underscore its growing importance in clinical genetics.

Based on the presence of mobile element *Alu* insertion in exon 4 of the *RP1* gene, it is believed that normal protein production is disrupted due to a frameshift mutation [8]. Frameshift mutations might alter the reading frame of the gene, leading to dysfunctional or truncated protein. The allele having this nonsense variant is considered as a null allele in inherited retinal diseases. In combination with another pathogenic variant, it can cause RP [7]. *Alu* insertions may result in a non-functional protein or misregulated gene expression, contributing to pathogenesis in a distinct way compared to other *RP1* mutations. Previously known mutations of the *RP1* gene include missense mutations, nonsense mutations, frameshift mutations, or insertion/deletion changes caused by single nucleotide substitutions in exon 4 [22]. *Alu* element insertion in RP1 differs from other mutations in terms of retrotransposition and its ability to disrupt splicing, silencing, or altering protein-coding sequences in a unique way. The specific molecular mechanisms of *Alu* insertion include RNA-mediated retrotransposition, integration into the genome, and the consequent disruption of the normal function of the *RP1* gene [23].

However, *Alu* element insertion in the *RP1* gene is often undetected in conventional targeted NGS [6,9] despite its high frequency [6]. Therefore, it is necessary to check whether *Alu* element insertion is present or not by performing additional analysis using WGS, especially in cases having the *RP1* gene with a pathogenic variant detected previously. In addition, if *Alu* element insertion is identified in a patient previously misinterpreted as having a novel autosomal dominant allele, modification to the classification as recessive inheritance may be necessary [9].

The three cases introduced in this study had no definite similarities in their clinical characteristics or their ophthalmological findings of RP. The involvement of macular area was different between cases—one involving diffuse peripheral retina sparing the posterior pole and the others involving the macula. Also, while central vision remained similar at 20/32 for Snellen visual acuity in the case sparing macular involvement, visual deterioration was seen in other cases. Since the number of patients was too small in this study, whether the presence of *Alu* element insertion would affect the clinical outcome of the disease for RP patients with *RP1* mutation is currently unclear. Further collection of more patients is needed to study their common clinical characteristics and analyze genotype–phenotype correlations in RP patients with *RP1* gene mutation having a compound heterozygous *Alu* element insertion.

In conclusion, *Alu* element insertion in the *RP1* gene could be a genetic cause of autosomal recessive RP patients with compound heterozygous variants. Through WGS, the identification of this pathogenic variant is possible, even if this variant is missed in standard targeted NGS.

## Figures and Tables

**Table 1:** Clinical characteristics and genetic findings of three patients with retinitis pigmentosa confirmed to have compound heterozygous variants in the *RP1* gene consisting of *Alu* element insertion newly detected by whole genome sequencing.

Case No.	Sex	Age at Symptom Onset	Age at Diagnosis	Age at Genetic Examination	Best-Corrected Visual Acuity	Nucleotide Change (Protein Change)	Zygosity
1	Male	10s	16	26	RE 20/32	c.4196delG (p.C1399 LeufsTer5)	Compound heterozygous
					LE 20/32	Alu element insertion	
2	Male	20s	37	40	RE 20/200	c.6181del (p.Ile2061fs12)	Compound heterozygous
					LE 20/63	Alu element insertion	
3	Male	10s	27	27	RE 20/50	c.6181del (p.Ile2061fs12)	Compound heterozygous
					LE 20/200	Alu element insertion	

## Data Availability

The datasets generated during and/or analyzed during the current study are available from the corresponding author on reasonable request.
